# Congenital urinary tract dilation: when is it clinically significant and when should it prompt intervention?

**DOI:** 10.3389/fped.2026.1757626

**Published:** 2026-04-30

**Authors:** Gunes Isik, Cemil Oktay

**Affiliations:** 1 Pediatric Nephrology, Adiyaman University, Adiyaman, Turkiye; 2 Radiology, Adiyaman University Faculty of Medicine, Adiyaman, Turkiye

**Keywords:** children, clinical prognostic factors, congenital urinary tract dilation, surgery, ultrasonography

## Abstract

**Background:**

Urinary tract dilation is the most common urinary tract anomaly detected on prenatal ultrasonography. Postnatal follow-up, the necessity of advanced imaging, and indications for surgical intervention remain confusing and subject to ongoing debate.

**Objectives:**

Our aim is to evaluate the etiology, clinical and radiological characteristics of congenital urinary tract dilation, and assess the timing and outcomes of spontaneous resolution, advanced imaging, surgical interventions, and postoperative follow-up.

**Methods:**

This retrospective study evaluated the etiology, clinical and radiological features, and outcomes of congenital urinary tract dilation in children at Adiyaman University Pediatric Nephrology Clinic between November 2021 and 2023. Urinary tract dilation was classified by ultrasonographic anteroposterior (AP) diameter on postnatal urinary system ultrasonography as normal (<10 mm), mild–moderate (10–15 mm), or severe (>15 mm).

**Results:**

Among 341 patients (71.3% male), 36.2% had severe and 63.8% had mild-to-moderate urinary tract dilation. Mean AP diameter was 14.4 ± 7.0 mm. Urinary tract dilation resolved in 96.2% of patients, typically within 3–44 months (mean 11 ± 6.3). Surgery was required in 22.6% of patients, mostly for ureteropelvic junction obstruction and vesicoureteral reflux, and was significantly more common in severe cases (56.1% vs. 3.7%, *p* < 0.001). Severe urinary tract dilation was significantly associated with family history of congenital anomalies of the kidney and urinary tract, history of urinary tract infection (UTI), reduced kidney function, and abnormal dimercaptosuccinic acid renal scintigraphy findings (*p* < 0.001). ROC analysis demonstrated high predictive performance (area under the receiver operating characteristic curve (AUC) = 0.936, 95% confidence interval: 0.908–0.963; *p* < 0.001). An AP diameter cutoff value of 17 mm predicted surgical intervention, with 88.3% sensitivity and 84.1% specificity.

**Conclusion:**

An AP diameter ≥17 mm, decreased differential kidney function, UTI history, and family history of congenital anomalies of the kidney and urinary tract are strong indicators for surgical intervention.

## Introduction

Urinary tract dilation (UTD) is the most common urinary tract anomaly detected on prenatal ultrasonography (US) and is frequently evaluated postnatally due to its accessibility and high anatomical resolution (1–3). Observed in 0.5%–1% of pregnancies, UTD often resolves spontaneously within the first year; however, 4%–15% of cases require surgical intervention (4, 5). UTD is an important predictor of congenital anomalies of the kidney and urinary tract (CAKUT), including posterior urethral valves, high-grade congenital vesicoureteral reflux (VUR), ureteropelvic junction obstruction (UPJO), and ureterovesical junction obstruction (UVJO), which vary in severity and may impair kidney function (1, 4, 6). Early identification of obstructive uropathies is critical, as CAKUT is the leading cause of pediatric kidney failure, associated with significantly reduced survival and long-term kidney complications (7).

In patients with severe UTD with UPJO, the indication for surgery is determined using diuretic renography. The most prevalent postnatal diagnoses include non-refluxing UTD (caused by UPJO) (10%–30%), primary VUR (10%–20%), primary non-refluxing megaureter (5%–10%), and ureterocele (5%) (8–11). Primary surgical indications are thinning of the kidney parenchyma, a differential renal function (DRF) below 40%, a decline of 10% or more in kidney function during follow-up, and recurrent urinary tract infections (9, 10). The frequency of VUR has been reported in 7−24% of congenital UTD cases. The association between high-grade VUR and urinary tract infection (UTI) is well known (12). In patients with UTD, voiding cystourethrography (VCUG) is primarily indicated based on ultrasonographic findings such as bilateral dilation, abnormal bladder appearance in male newborns, or associated ureteral dilation, to exclude posterior urethral valves. During follow-up, VCUG should be performed in patients with UTD who develop recurrent febrile urinary tract infections to identify congenital vesicoureteral reflux (6, 13).

In congenital UTD, a key challenge is determining which patients need long-term follow-up, advanced imaging, or surgery, and which cases will resolve spontaneously. Clear protocols are needed to guide monitoring, avoid unnecessary tests and treatments, reduce parental anxiety, minimize radiation exposure, and lower healthcare costs (1, 14).

This study aimed to determine which patients with congenital UTD require advanced imaging and to identify indicators for surgical intervention. It also evaluated etiologic factors, imaging findings, and postoperative outcomes in a large patient cohort, providing practical guidance for clinicians.

## Material and methods

A retrospective evaluation was conducted on patients aged 0 months to 18 years who were admitted to the Pediatric Nephrology Clinics of Adiyaman University School of Medicine with a diagnosis of congenital UTD between 1 November 2021 and 1 November 2023. The primary inclusion criterion was the presence of an elevated anteroposterior (AP) renal pelvic diameter on postnatal urinary tract ultrasonography, consistent with the UTD classification system. All patients presenting to the pediatric nephrology outpatient clinic were retrospectively screened. Patient selection was based on hospital medical records, and patients diagnosed with congenital hydronephrosis, as identified by diagnostic coding, were included in the study. Patients were classified according to the UTD consensus system based on the timing of diagnosis. In antenatally diagnosed cases, urinary tract dilation was defined using the anteroposterior renal pelvic diameter (APRPD) measured after 32 weeks of gestation, with an APRPD ≥7 mm considered pathological, in accordance with UTD antenatal criteria. In postnatally diagnosed cases, ultrasonographic assessment was interpreted within the UTD postnatal framework. Consistent with this system, APRPD thresholds of 10–15 and ≥15 mm were used as key cutoffs reflecting increasing risk categories. APRPD-based grouping was applied for severity assessment and statistical analyses (15). In severe UTD, monthly US was performed, and resolution was defined as an APRPD <10 mm on two consecutive scans in non-operated cases, or when severe UTD improved to mild/moderate UTD after surgery. Demographic data, family history, imaging results [US, dimercaptosuccinic acid renal scintigraphy (DMSA), technetium-99 m mercaptoacetyltriglycine renal scintigraphy (MAG3), diethylenetriamine pentaacetic acid renal scintigraphy (DTPA), and VCUG], and surgical findings were recorded. A bottom-up approach was used for VCUG and MAG3/DTPA. Patient age was recorded at the time of diagnosis. History of UTI referred to episodes of UTI documented during the follow-up period. UTI was diagnosed based on a combined evaluation of clinical symptoms and signs, together with urinalysis and urine culture results. Confirmation required significant growth of a single pathogen in catheterized urine samples (16–20). Surgical referral for UPJO was indicated by an initial split kidney function <40%, a decline >10% on follow-up, progressive increase in APRPD, or the presence of flank pain, pyelonephritis, or obstructive calculi. Interventions included pyeloplasty for UPJO and ureterostomy, reimplantation, or ureterovesicostomy for UVJO. In UPJO, a double-J catheter was inserted after pyeloplasty, removed after 1 month, and evaluated by control ultrasonography (9). Surgery for VUR was indicated in cases of recurrent UTI despite antibiotic prophylaxis, progressive kidney damage or scarring on DMSA scans, impaired kidney growth, or non-compliance with prophylaxis. Surgical options included endoscopic antireflux procedures (STING) or ureteral reimplantation. Antibiotic prophylaxis was given only to patients with recurrent UTI or high-grade VUR prior to surgery (11–13).

### Statistical analysis

All statistical analyses were performed using SPSS version 26.0 (IBM Corp., Armonk, NY, USA). Continuous variables are presented as mean ± standard deviation or as median (minimum–maximum), as appropriate for data distribution. Categorical variables are expressed as frequencies and percentages. Between-group comparisons of categorical variables were performed using the chi-square test. Multivariable logistic regression analysis was conducted to identify independent predictors of surgical requirement. Variables with clinical relevance were entered into the model using the Enter method, and odds ratios (ORs) with 95% confidence intervals (CIs) were calculated. Receiver operating characteristic (ROC) curve analysis was subsequently applied to variables identified as independent predictors in order to evaluate their discriminative performance and to determine optimal cutoff values. ROC analysis was used to predict the presence of an anatomical abnormality requiring surgery according to the AP pelvic diameters of the hydronephrotic kidneys of patients. Statistical significance was set at *p* < 0.05.

## Results

The present study included 341 children with congenital UTD, of whom 71.3% were male. Mild-to-moderate dilation was observed in 63.8% of patients, while 36.2% had severe dilation. Associated anomalies were identified in a substantial proportion of cases, most commonly VUR (28%) and UPJO (9.4%). UTD resolution, defined as overall radiological improvement on follow-up ultrasonography, was observed in 96.2% of patients, most often within the first year of life. In surgically treated cases, resolution was characterized by regression from severe to mild or moderate urinary tract dilatation, whereas in non-operated patients, resolution was defined as an APRPD of <10 mm. Among patients with severe dilation, regression to mild/moderate occurred within a mean of 8.7 ± 5.1 months, including those who underwent surgical intervention (Table 1). Surgical treatment was required in 22.6% of patients, most commonly for UPJO and VUR, with pyeloplasty (50.6%) and subureteric injection (40.3%) being the predominant procedures. On DMSA scintigraphy, 19.3% of patients showed kidney scarring, 48.7% had decreased function, and 14.0% had both scarring and functional loss. Loss of kidney function developed within a mean of 7.6 ± 5.8 months. VUR was identified in 31.0% of patients undergoing VCUG, and MAG-3 scintigraphy revealed obstructive patterns consistent with UPJO in a subset of cases; no crossing vessels were observed.

**Table 1:** Descriptive characteristics of the patients (*n* = 341).

	*n*	%
Gender	*n*	%
Female	98	28.7
Male	243	71.3
History of UTI	*n*	%
Yes	101	29.6
No	240	70.4
History of UTD in the sibling	*n*	%
Yes	19	5.6
No	322	94.4
	Mean ± SD	Med (min–max)
Variable		
Age (mo)	5,6 ± 4,4	4 (1–30)
Body weight (kg)	6.84 ± 2.16	6,700 (2,400–15,000)
Height (cm)	63.6 ± 8.7	62 (47–98)
Laterality of UTD	*n*	%
Left kidney	181	53.1
Right kidney	64	18.8
Bilateral	96	28.2
Severity of UTD	*n*	%
Mild–moderate	217	63.8
Severe	123	36.2
Resolution of UTD	*n*	%
No	13	3.8
Yes	328	96.2

UTI, urinary tract infection; UTD, urinary tract dilation.

UTI and a positive family history of congenital urinary tract dilation were both significantly more frequent in patients with severe UTD (52% vs. 16.6% and 12.2% vs. 1.8%, respectively; *p* < 0.001). Surgical intervention was required significantly more often in the severe UTD group compared with the non-severe UTD group (56.1% vs. 3.7%, *p* < 0.001). Functional kidney impairment was also more prevalent in severe UTD, with abnormal DMSA findings observed more frequently. Normal DMSA scans were less common in severe cases (35.8% vs. 72.5%, *p* = 0.001), while reduced kidney function was significantly higher (58.7% vs. 22.5%, *p* < 0.001). Obstructive patterns on diuretic renography were identified in 46.6% of patients with severe UTD, whereas no obstructive findings were detected in the non-severe UTD group (*p* < 0.001) (Table 2).

**Table 2:** Imaging, clinical characteristics, and treatment modalities of the patients (*n* = 341).

UTD classification						
	Mild-moderate, *n* (%)	Severe, *n* (%)	*p*	Conservative treatment, *n* (%)	Surgical treatment, *n* (%)	*p*
Gender						
Female	66 (30.4)	32 (26)	0.390	75 (28.4)	23 (29.9)	0.803
Male	151 (69.6)	91 (74)		189 (71.6)	54 (70.1)	
History of UTI	*n (%)*	*n (%)*	*p*	*n (%)*	*n (%)*	*p*
Yes	36 (16.6)	64 (52)	<0.001	47 (17.8)	54 (70.1)	<0.001
No	181 (83.4)	59 (48)		217 (82.2)	23 (29.9)	
History of UTD in sibling(s)	*n (%)*	*n (%)*	*p*	*n (%)*	*n (%)*	*p*
Yes	4 (1.8)	15 (12.2)	<0.001	4 (1.5)	15 (19.5)	<0.001
No	213 (98.2)	108 (87.8)		260 (98.5)	62 (80.5)	
Surgical treatment	*n (%)*	*n (%)*	*p*			
No	209 (96. .3)	54 (43.9)	<0.001			
Yes	8 (3.7)	69 (56.1)				
DMSA renal scan findings (*n* = 150)	*n (%)*	*n (%)*	*p*	*n (%)*	*n (%)*	*p*
Normal	29 (72.5)	39 (35.8)	0.001	67 (91.8)	2 (2.6)	-
Decreased kidney function	7 (17.5)	45 (41.3)		3 (4.1)	49 (63.6)	
Kidney scarring	2 (5)	6 (5.5)		2 (2.7)	6 (7.8)	
Loss of kidney function + kidney scarring	2 (5)	19 (17.4)		1 (1.4)	20 (26)	
Kidney scarring on DMSA scans (*n* = 150)	*n (%)*	*n (%)*	*p*	*n (%)*	*n (%)*	
Yes	4 (10)	25 (22.9)	0.125	3 (4.1)	26 (33.8)	<0.001
No	36 (90)	84 (77.1)		70 (95.9)	51 (66.2)	
Loss of kidney function on DMSA renal scan (*n* = 150)	*n (%)*	*n (%)*	*p*	*n (%)*	*n (%)*	*p*
No	31 (77.5)	45 (41.3)	<0.001	69 (94.5)	8 (10.4)	<0.001
Yes	9 (22.5)	64 (58.7)		4 (5.5)	69 (89.6)	
VUR on VCUG (*n* = 129)	*n (%)*	*n (%)*	*p*	*n (%)*	*n (%)*	*p*
No	24 (77.4)	65 (66.3)	0.347	52 (94.5)	37 (50)	<0.001
Yes	7 (22.6)	33 (33.7)		3 (5.5)	37 (50)	
MAG-3/DTPA renal scan findings (*n* = 113)	*n (%)*	*n (%)*	*p*	*n (%)*	*n (%)*	*p*
Right-sided obstructive uropathy	0 (0)	13 (15.4)	-	0 (0)	13 (27.6)	-
Left-sided obstructive uropathy	0 (0)	26 (31)		0 (0)	26 (55.3)	
Right-sided partial obstruction	6 (20.7)	9 (10.7)		13 (19.7)	2 (4.2)	
Left-sided partial obstruction	10 (34.5)	31 (36.9)		40 (60.6)	1 (2.3)	
Normal	12 (41.4)	5 (6)		12 (18.2)	5 (10.6)	
Bilateral partial obstruction	1 (3.4)	0 (0)		1 (1.5)	0 (0)	
MAG-3/DTPA renal scan findings (*n* = 113)	*n (%)*	*n (%)*	*p*	*n (%)*	*n (%)*	*p*
Normal	29 (100)	45 (53.6)	<0.001	66 (100)	8 (17)	<0.001
Abnormal	0 (0)	39 (46.4)		0 (0)	39 (83)	
Loss of kidney function on DMSA renal scan (*n* = 150)	*n (%)*	*n (%)*	*p*	*n (%)*	*n (%)*	*p*
No	31 (77.5)	45 (41.3)	<0.001	69 (94.5)	8 (10.4)	<0.001
Yes	9 (22.5)	64 (58.7)		4 (5.5)	69 (89.6)	
VUR on VCUG (*n* = 129)	*n (%)*	*n (%)*	*p*	*n (%)*	*n (%)*	*p*
No	24 (77.4)	65 (66.3)	0.347	52 (94.5)	37 (50)	<0.001
Yes	7 (22.6)	33 (33.7)		3 (5.5)	37 (50)	
MAG-3/DTPA renal scan findings (*n* = 113)	*n (%)*	*n (%)*	*p*	*n (%)*	*n (%)*	*p*
Right-sided obstructive uropathy	0 (0)	13 (15,4)	-	0 (0)	13 (27.6)	-
Left-sided obstructive uropathy	0 (0)	26 (31)		0 (0)	26 (55.3)	
Right-sided partial obstruction	6 (20.7)	9 (10.7)		13 (19.7)	2 (4.2)	
Left-sided partial obstruction	10 (34.5)	31 (36.9)		40 (60.6)	1 (2.3)	
Normal	12 (41.4)	5 (6)		12 (18.2)	5 (10.6)	
Bilateral partial obstruction	1 (3.4)	0 (0)		1 (1.5)	0 (0)	
MAG-3/DTPA renal scan findings (*n* = 113)	*n (%)*	*n (%)*	*p*	*n (%)*	*n (%)*	*p*
Normal	29 (100)	45 (53.6)	<0.001	66 (100)	8 (17)	<0.001
Abnormal	0 (0)	39 (46.4)		0 (0)	39 (83)	

VCUG, voiding cystourethrography; DMSA, dimercaptosuccinic acid renal scintigraphy; MAG-3, technetium-99m mercaptoacetyltriglycine renal scintigraphy; DTPA, diethylenetriamine pentaacetic acid renal scintigraphy.

**Figure 1:**
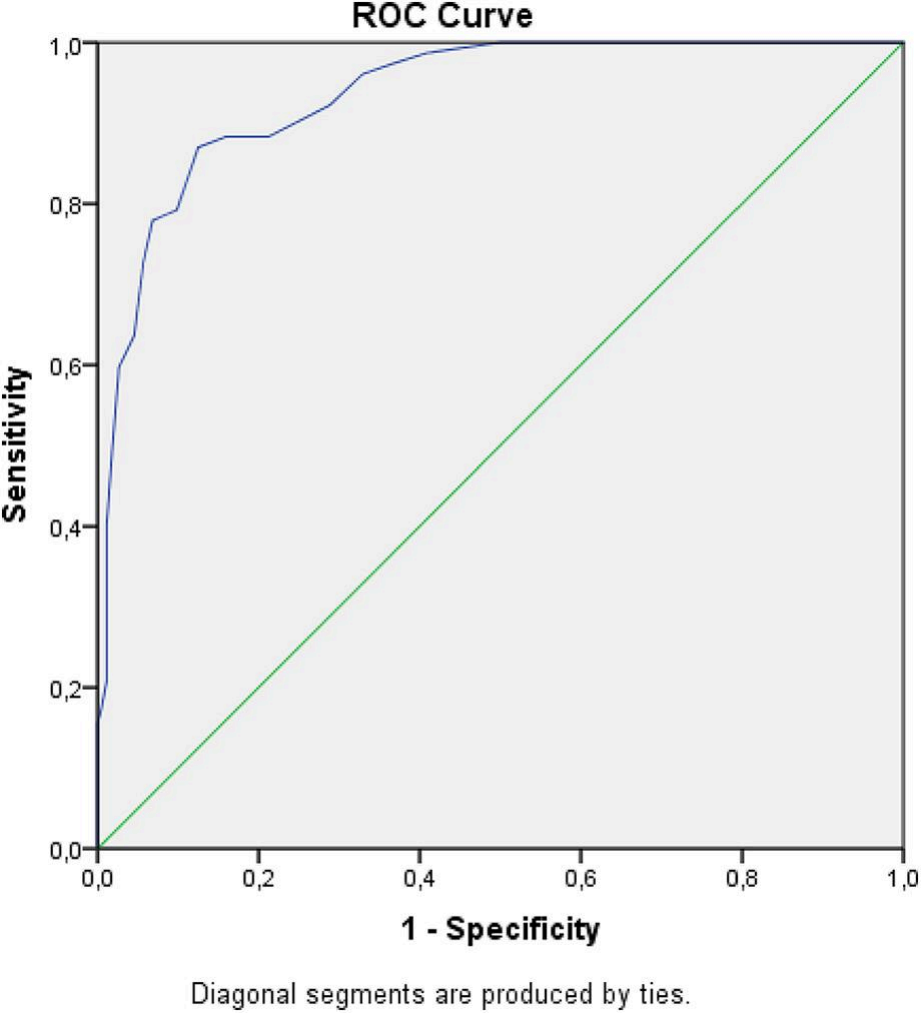
Predictability of anatomical abnormalities according to pelvic AP diameters of hydronephrotic kidneys (ROC analysis) [AUC: 0.936 (0.908–0.963) *p*<0.001].

Surgically treated patients showed significantly higher rates of UTI (70.1% vs. 17.8%, *p* < 0.001) and a positive sibling history of CAKUT (19.5% vs. 1.5%, *p* < 0.001). Kidney scarring on DMSA was more frequent in surgically treated patients (33.8% vs. 4.1%, *p* < 0.001), as was functional loss (89.6% vs. 5.5%, *p* < 0.001). VUR was detected in 50% of surgical patients compared with 5.5% of those managed conservatively (*p* < 0.001). Obstructive patterns on diuretic renography were observed in 83% of surgical patients, whereas none were identified in the non-surgical group (*p* < 0.001) (Table 2). Multivariable logistic regression analysis demonstrated that history of urinary tract infection, sibling history of CAKUT, and AP diameter were independently associated with an increased likelihood of surgical intervention, whereas sex was not a significant independent predictor. The results of the regression analysis are summarized in Table 3. Based on the findings of the multivariable logistic regression analysis, ROC curve analysis was performed for AP diameter to evaluate its discriminative ability in predicting surgical requirement. ROC analysis demonstrated that APRPD was predictive of anatomical abnormalities requiring surgical intervention. An APRPD diameter cutoff value of 17 mm was identified, yielding an AUC of 0.936 (95% CI: 0.908–0.963; *p* < 0.001). At this threshold, sensitivity and specificity were 88.3% and 84.1%, respectively, with a positive predictive value of 61.8% and a negative predictive value of 96.1% (Figure 1).

**Table 3:** Logistic regression analysis of factors predicting the need for surgery.

Variable	*β* coefficient	Standard error	Wald *χ*^2^	Odds ratio (OR)	95% CI		*p*-Value
Female sex (reference category: male)	0.661	0.468	2.000	1.938		0.775–4.846	0.157
History of UTI (reference category: no)	1.580	0.435	13.198	4.856		2.070–11.388	<0.001
Sibling history (reference category: no)	1.952	0.702	7.728	7.041		1.778–27.879	0.005
AP diameter	0.364	0.048	58.809	1.440		1.312–1.580	<0.001

Multivariable logistic regression analysis was performed using the Enter method. The dependent variable was surgical requirement (0 = no, 1 = yes).

*N* = 341; Nagelkerke *R*^2^ = 0.712.

OR, odds ratio; CI, confidence interval; UTI, urinary tract infection; AP, anteroposterior.

## Discussion

Congenital UTD represents one of the most frequent indications for referral to pediatric nephrology and urology outpatient clinics. US constitutes the cornerstone of both initial diagnostic evaluation and longitudinal follow-up, while advanced imaging modalities should be reserved for carefully selected cases based on clinical and radiological risk stratification. The present study aimed to characterize the radiological course of congenital UTD and to delineate the clinical and radiological indicators associated with failure of spontaneous resolution and the requirement for surgical intervention.

In a previous study evaluating 193 conservatively managed newborns, spontaneous resolution of UTD within the first year was observed in 73% of cases with a renal pelvis AP diameter of 5–15 mm (21). In another study by Elmaci and Dönmez, where 276 patients with AP diameters of renal pelvises measuring ≤20 mm were evaluated, complete resolution was achieved in 71.7% of the patients at 16 months of follow-up (22). In our cohort, renal pelvic AP diameters ranged from 6 to 39 mm, and overall radiological improvement was observed in 96.2% of patients during follow-up. This reflects both conservative improvement and postoperative regression observed during follow-up. Severe UTD regressed to mild or moderate grades within a mean follow-up period of 8.7 months, which is consistent with previous reports evaluating postoperative outcomes (23–26).

Renal pelvic AP diameter emerged as an important predictor for further intervention. While different thresholds have been proposed in the literature, values ranging between 15 and 16 mm have been suggested as indicators for closer evaluation (21, 22, 25–27). In our study, ROC analysis identified an AP diameter cutoff of 17 mm as a significant predictor for the presence of anatomical abnormalities associated with the need for surgical intervention [AUC 0.936 (0.908–0.963), *p* < 0.001]. However, surgical decisions were not based on AP diameter alone. Clinical characteristics—such as age, sex, history of urinary tract infection, kidney scarring or functional loss on DMSA scans, and obstructive findings on diuretic renography—were also taken into account. Therefore, this cutoff value should be interpreted as a warning sign prompting closer follow-up and further evaluation, rather than an absolute indication for surgery.

In accordance with the existing literature, the presence of renal cortical scarring on DMSA scintigraphy was identified as a significant risk factor for underlying anatomical abnormalities necessitating surgical intervention, particularly in the context of vesicoureteral reflux (11, 12). Furthermore, history of urinary tract infection, reduced differential renal function, obstructive drainage patterns on diuretic renography, and family history of congenital UTD were all significantly more prevalent among patients with severe UTD, collectively reinforcing the importance of a comprehensive clinical and radiological evaluation in this population.

Ultrasonography remains the cornerstone of diagnosis and follow-up in patients with UTD, and optimal management requires a multidisciplinary approach involving pediatricians, pediatric nephrologists, pediatric urologists, and pediatric surgeons. In line with the 2025 American Academy of Pediatrics recommendations, our findings support a risk-stratified approach in which ultrasonographic surveillance is sufficient for most patients, while advanced imaging is reserved for those with high-risk clinical or radiological features (13). This strategy supports individualized, guideline-based decision-making and may help reduce unnecessary investigations.

The findings of the present study support a risk-stratified follow-up strategy in children with congenital UTD, for whom ultrasonographic surveillance remains adequate in the majority of cases. Nevertheless, the identification of warning signs—including an elevated APRPD, declining DRF, or documented history of urinary tract infections—should prompt more intensive surveillance and the timely incorporation of advanced imaging to facilitate individualized clinical decision-making, while avoiding both delayed intervention and unnecessary diagnostic procedures.

An AP diameter cutoff of 17 mm should be interpreted as a warning for close follow-up, detailed imaging, and further evaluation if necessary, rather than as an absolute indication for surgery. Validation in multicenter, prospective studies is needed to assess its broader applicability and generalizability.

## Limitations

The retrospective, single-center design of this study limits the generalizability of the findings. The retrospective nature of data collection is susceptible to information bias, as the completeness and consistency of clinical records cannot be fully verified. The absence of a standardized prospective follow-up protocol may have introduced variability in the timing and frequency of clinical and radiological assessments across patients. Another limitation of our study is the lack of assessment of factors such as male circumcision and bowel dysfunction on UTI risk. In addition, the proposed AP diameter cutoff should be interpreted with caution. Surgical decisions in this cohort were based on overall clinical assessment and imaging findings, with AP diameter constituting one of several contributing factors. Therefore, a degree of incorporation bias cannot be excluded, and the identified threshold may reflect institutional clinical decision-making rather than a universal surgical indication. Furthermore, reliance on administrative coding and tertiary-care referrals may have resulted in under-ascertainment of milder or asymptomatic cases, introducing potential ascertainment bias. Prospective multicenter validation is required before broader clinical application. Despite these limitations, this study has notable strengths. It includes a large, well-characterized pediatric cohort evaluated at a tertiary care center serving a region with a high birth rate, providing robust real-world data from a clinically relevant population. The substantial sample size enhances the statistical power of the analysis and allows for a more comprehensive evaluation of clinical characteristics and outcomes. Future multicenter, prospective studies incorporating estimated glomerular filtration rate (eGFR) monitoring, postoperative kidney function assessment, and long-term follow-up of CAKUT would provide important insights into the long-term progression and optimal management of congenital UTD.

## Conclusions

In our study, renal pelvic AP diameters ≥17 mm, a decrease in DRF on diuretic renography, history of UTI, and family history of congenital UTD were identified as important warning signs for patients who may require surgical intervention. These patients should be monitored more closely and evaluated with particular care.

## Data Availability

The original contributions presented in the study are included in the article/Supplementary Material, further inquiries can be directed to the corresponding author.
